# A cleanroom sleeping environment’s impact on markers of oxidative stress, immune dysregulation, and behavior in children with autism spectrum disorders

**DOI:** 10.1186/s12906-015-0564-0

**Published:** 2015-03-19

**Authors:** Scott Faber, Gregory M Zinn, Andrew Boggess, Timothy Fahrenholz, John C Kern, HM Skip Kingston

**Affiliations:** Medicine, The Children’s Institute, 1405 Shady Avenue, Pittsburgh, PA 15217 USA; Chemistry and Biochemistry, Duquesne University, 600 Forbes Avenue, Pittsburgh, PA 15282 USA; Mathematics and Computer Science, Duquesne University, 600 Forbes Avenue, Pittsburgh, PA 15282 USA

**Keywords:** Autism, Behavioral rating scales, Cleanroom, Glutathione, Immune dysregulation, Oxidative stress

## Abstract

**Background:**

An emerging paradigm suggests children with autism display a unique pattern of environmental, genetic, and epigenetic triggers that make them susceptible to developing dysfunctional heavy metal and chemical detoxification systems. These abnormalities could be caused by alterations in the methylation, sulfation, and metalloprotein pathways. This study sought to evaluate the physiological and behavioral effects of children with autism sleeping in an International Organization for Standardization Class 5 cleanroom.

**Methods:**

Ten children with autism, ages 3–12, slept in a cleanroom for two weeks to evaluate changes in toxin levels, oxidative stress, immune dysregulation, and behavior. Before and after the children slept in the cleanroom, samples of blood and hair and rating scale scores were obtained to assess these changes.

**Results:**

Five children significantly lowered their concentration of oxidized glutathione, a biomarker of oxidative stress. The younger cohort, age 5 and under, showed significantly greater mean decreases in two markers of immune dysregulation, CD3% and CD4%, than the older cohort. Changes in serum magnesium, influencing neuronal regulation, correlated negatively while changes in serum iron, affecting oxygenation of tissues, correlated positively with age. Changes in serum benzene and PCB 28 concentrations showed significant negative correlations with age. The younger children demonstrated significant improvements on behavioral rating scales compared to the older children. In a younger pair of identical twins, one twin showed significantly greater improvements in 4 out of 5 markers of oxidative stress, which corresponded with better overall behavioral rating scale scores than the other twin.

**Conclusions:**

Younger children who slept in the cleanroom altered elemental levels, decreased immune dysregulation, and improved behavioral rating scales, suggesting that their detoxification metabolism was briefly enhanced. The older children displayed a worsening in behavioral rating scale performance, which may have been caused by the mobilization of toxins from their tissues. The interpretation of this exploratory study is limited by lack of a control group and small sample size. The changes in physiology and behavior noted suggest that performance of larger, prospective controlled studies of exposure to nighttime or 24 hour cleanroom conditions for longer time periods may be useful for understanding detoxification in children with autism.

**Trial registration:**

Clinical Trial Registration Number NCT02195401 (Obtained July 18, 2014).

## Background

Children with autism spectrum disorders (ASDs) display dysfunction in the areas of expressive language production and quality, socialization ability, and control of excessive repetitive thoughts and behaviors [1]. Over the past two decades, worldwide incidence and prevalence of these disorders have increased; however, scholarly opinion varies as to whether this rise is due to improved awareness, better age-related screening efforts, higher quality and quantity of diagnostic centers, an expansion of the spectrum definition, the types of study methods used, or a true escalation in the incidence and prevalence of autism [2-8]. Although much research has focused on finding a genetic cause of autism, it has not been successful in explaining a majority of the diagnoses. In children with ASDs, only 21% display genetic differences from the typically sequenced human DNA [9]. Copy number variants and losses have been strongly associated with the development of autism [10], potentially indicating a mechanism for genetic/environmental interaction.

### Effects of toxin exposure

A recent review noted the possible contributions of toxin exposure during gestation and early childhood to the development of autism [11]. Emerging literature on the exposome, which measures environmental, extracellular, and intracellular influences on the expression of the genome, strongly suggests that adverse neurological outcomes can be created by pre- and postnatal exposure to toxins [12]. With the lack of success in finding a purely genetic cause for autism, an emerging paradigm suggests that one of the causes of the increased prevalence of ASDs could be difficulties in heavy metal and chemical detoxification [13]. Multiple studies have previously correlated proximity to coal fired power plants, pesticide-rich agricultural fields, known toxic chemical sites, and traffic-related air pollution with the prevalence of ASDs [14-18]. It has been hypothesized that difficulties in detoxification can cause oxidative stress [19-23] and immune dysregulation [24-28], leading to or exacerbating ASDs phenotypic presentation [29,30].

Exposure to environmental toxins, including heavy metals and chemicals, have been shown to negatively affect systems of detoxification involving glutathione [31-33] and metallothionein [34,35], of which both zinc and selenium are key cofactors [36]. Glutathione is the central antioxidant responsible for environmental toxin detoxification [37] and has been used as a biomarker of oxidative stress to evaluate the methylation and sulfation systems [38,39]. The metallothionein system is also necessary for heavy metal detoxification [40] and can be assessed by the plasma zinc/serum copper ratio [35,41]. Studies have shown that total glutathione, reduced/oxidized glutathione ratio, and zinc/copper ratio are often lower in children with ASDs [35,38,39,41]. Lower amounts of total glutathione, triggered by increased environmental toxin exposure, have been shown to also affect the immune system of children with autism, as evidenced by lower natural killer (NK) cell activity [42].

Difficulties in detoxification of heavy metals and chemicals can lead to autoimmunity [43,44] and immune dysregulation [24,25,27]. Forms of autoimmunity which use NK cells to attack brain tissue [45] may contribute to the under-connectivity between distant areas of the brains of children with autism [46]. Certain markers of immunological regulation and function [47-49], such as the T cell helper/suppressor (CD4:CD8) ratio and CD4% [50], are indicative of dysregulated innate and humoral immunity [51]. Both the CD4:CD8 ratio and CD4% have been shown to correlate with performance on the Autism Behavior Checklist [50], indicating a link between immunological function and phenotypic presentation. In addition, altered cytokine profiles in the immune systems of children with ASDs have been connected to behavioral instabilities [52]. Immune dysfunction has been found in 15% to 60% of children with ASDs [53], and it was recently shown that, when compared with controls, children with ASDs exhibited greater evidence of increased detoxification difficulties, oxidative stress, and immune dysregulation [54].

### Cleanrooms and detoxification

Given the emerging evidence connecting poor detoxification of environmental toxins to the biomedical and clinical presentation of ASDs, an intervention that temporarily decreases environmental exposure may positively impact phenotypic presentation [55]. Concentrations of fine particles and gaseous chemicals can be reduced in cleanrooms by using high efficiency particulate air (HEPA) filters and activated carbon. Individuals who have spent time in a cleanroom have presented reduced symptoms associated with immunological response to IgE mediated allergies and mold [56-58]. These unique allergic and immunological responses cause the release of mast cell mediators that can affect the central nervous system resulting in detrimental behavioral effects in certain children with autism [59].

The application of cleanroom technology in the treatment of children with ASDs has been previously undertaken [60-63]. These children improved significantly and obviously in areas of expressive language, socialization, and obsessiveness after the first several weeks of detoxification in their cleanrooms, and appeared to maintain these gains even once removed from the clean environment [60-63]. It was discovered that children with milder forms of autism appeared to improve when exposed to lesser levels of environmental cleanliness, while children with more severe autism needed to be fully removed from environmental toxicity before gains could be seen. These studies suggest that a cleanroom, which reduces the burden on detoxification systems, may lead to decreased oxidative stress and improved immunological and neurological functioning in children with autism. Multiple studies have demonstrated the importance of early treatment interventions [64-66]. It has been shown that younger children, under age 6, with autism display greater and more rapid response to intensive treatments, and there may be a crucial time interval for intervention before the end of early life developmental plasticity [64].

This study sought to evaluate the effects of a cleanroom sleeping environment on toxin levels and markers of oxidative stress, immune dysregulation, and behavioral presentation in ten children with autism, 3 to 12 years of age. These effects were evaluated by quantifying changes in blood toxin concentrations, glutathione levels, and markers of immune function, and determining the correlation between age and improvement in these markers.

## Methods

After obtaining approval from the Institutional Review Board (IRB) of Duquesne University (Pittsburgh, PA, USA, IRB #09-131), the cleanroom was created and the study was conducted at The Children’s Institute (TCI, Pittsburgh, PA, USA). Before enrolling in the study, consent was obtained from either a parent or legal guardian, and a pictorial document explaining the study was reviewed with each child.

### Subject enrollment and study design

Ten children, ages 3–12, with autistic disorder confirmed by a psychologist who is research certified to perform the Autism Diagnostic Observation Schedule (ADOS) [67] were recruited from the Neurodevelopmental Service of TCI. Eligible children had to have prior evidence of heavy metal and chemical toxicity and immune dysregulation seen through low plasma zinc/serum copper ratios and abnormal T and B cell subsets, respectively. Child #2 was unable to participate in the study because of the inability to complete a blood draw. The group intentionally contained two sets of identical twins (ages 5 and 12). Children were not eligible if they had severe behavioral dysregulation, uncontrolled seizure activity, or any other severe chronic medical condition, other than autism, that required frequent management by general pediatricians.

After signing the consent and being provided opportunity for child assent, each child and a parent spent two consecutive weeks sleeping in the cleanroom between May and October 2010. During the day, the child and parent went about their normal activities and therapies. The child and parent arrived at TCI and spent approximately ten hours each evening in the cleanroom while wearing silk or cotton clothes. A research assistant recorded each child’s sleep behaviors. The children and family’s adjustment to the study was monitored by a Family Advocate Psychologist who had no academic involvement in the study. Diet, medications, nutrients, and experiential services were held constant throughout the study.

### Cleanroom design and classification

The cleanroom was designed and built at TCI in a modified hospital room. Cleanroom modifications were made to the floors, walls, and ceiling tiles and the window was encased in a secondary transparent enclosure. HEPA filters were mounted in each of the four air intakes in the room. A Model 7512 Second Wind Air Purifier (Buffalo, NY, USA) was placed in the room and set to the lowest setting. The room was furnished with ash furniture sealed with water soluble polyurethane from Stevens Woodworks (Allison Park, PA, USA). The cleanroom bedding was purchased from Naturepedic (Chagrin, OH, USA). An ultraviolet water purification system was installed in the adjoining bathroom.

The cleanroom classification testing was performed using a Hach (Loveland, CO, USA) Met One HHPC-6 Handheld Airborne Particle Counter. Cleanroom classification was performed following International Organization for Standardization (ISO) standards using 0.5 μm particles [68].

### Blood draws, hair collection, and clinical testing

Within 24 hours before and after the two week period of sleeping in the cleanroom, approximately 30 milliliters of blood were drawn by a phlebotomist who specialized in working with children with developmental disabilities. The draws were completed at the child’s home and the blood was processed and stored within two hours. The blood was separated with a calibrated centrifuge and aliquoted for testing by Quest Diagnostics (Pittsburgh, PA, USA) and Duquesne University. In addition, a hair sample was obtained from the back of the head, and it was placed in a clean, sealable polyethylene bag for transport to Duquesne University for analysis. The Quest Diagnostics testing included plasma zinc; serum copper; T and B lymphocyte subsets; serum IgA, IgG, and IgM immunoglobulins; and complete blood count including differential and platelets. The Duquesne University testing included elemental analyses in serum, plasma, red blood cells (RBCs), and hair; total, oxidized, and reduced glutathione in RBCs; and benzene, toluene, and o-xylene along with selected polychlorinated biphenyls (PCBs) in serum. Before analysis, the serum, plasma, and RBCs to be tested by Duquesne University were stored at −80°C.

### Blood and hair elemental quantification

Blood elemental analysis was conducted in serum, plasma, and RBCs. For both before and after the cleanroom samples, two subsamples were prepared for each matrix and child. Each 0.2 gram subsample was digested with ARISTAR ULTRA nitric acid and ARISTAR ULTRA hydrochloric acid in a Milestone Inc. (Shelton, CT, USA) ETHOS 1 laboratory microwave system according to EPA Method 3052 [69]. Samples were stored in a cold room at 4°C until analysis. Total elemental analysis was performed according to EPA Method 6020B [70]. An additional separate analysis was performed using Isotope Dilution Mass Spectrometry (IDMS), a component of EPA Method 6800 [71] for 9 selected analytes (antimony, cadmium, chromium, copper, lead, mercury, molybdenum, selenium, and zinc) by spiking an isotopically enriched mixture of each analyte into the sample prior to microwave digestion. The analyses were performed using an Agilent (Santa Clara, CA, USA) 7700 Inductively Coupled Plasma-Mass Spectrometer operated in hydrogen, helium, and no gas modes. The instrument was tuned in all three analysis-modes in addition to determining the pulse/analog factor each day before analysis. An Agilent internal standard solution was used, and four replicates taken from each subsample. The data was taken from Agilent MassHunter software after analysis and exported into Microsoft Excel for further data processing. Standard Reference Material 1598a (Inorganic Constituents in Animal Serum, National Institute of Standards and Technology, Gaithersburg, MD, USA) was used to validate the sample preparation and analysis methods.

Hair elemental analysis was performed using EPA Method 3052 microwave sample preparation and EPA Method 6020B quantification. Sample preparation, analysis, storage, and quantification procedures followed which were identical to the blood elemental analysis procedure, except for using 0.1 gram per subsample. Certified Reference Material GBW09101b (Shanghai, China), Human Hair, was used as quality control for sample preparation and analysis.

### Organic toxin quantification

Organic compound quantification was performed on the volatile organic compounds (VOCs) benzene, toluene, and o-xylene using EPA Method 1624 [72] and selected PCBs (PCB-28, PCB-52, PCB-101, PCB-138, PCB-153, and PCB-180) using EPA Method 1625 [73]. Separate analyses were completed for VOC and PCB quantification, each requiring 200 μL of serum spiked with ^2^H labeled versions of each VOC or ^13^C labeled versions of each PCB. Solid-phase microextraction was performed on the headspace of the samples using a polydimethylsiloxane/divinylbenzene fiber for VOCs and polyacrylate fiber for the PCBs. An automated Gerstel (Linthicum, MD, USA) MultiPurpose Sampler II and Agilent 7890A Gas Chromatography and an Agilent 5975c quadrupole Mass Spectrometer were used for sample analysis and Agilent Chemstation for data analysis. Three independent samples were prepared for each sample, both before and after the cleanroom, with one analysis performed on each.

### Glutathione

The blood used for glutathione analysis was transported to Duquesne University on ice and shielded from light. The samples were aliquoted in an inert nitrogen atmosphere and then centrifuged at 2,000 relative centrifugal force (RCF) at 4°C for 15 minutes. The separated RBCs were aliquoted under inert atmosphere conditions and placed into a −80°C freezer for storage.

For total glutathione (tGSH) analysis, 30 milligrams (mg) of RBCs were combined with a known amount of enriched GSH and 300 mgs of approximately 10,000 microgram gram^−1^ (μg g^−1^) dithiothreitol for 20 minutes. Each sample was then treated with 1 mL of acetonitrile (ACN) to precipitate out the proteins, and the sample was centrifuged at 4°C at 5,000 RCF. The resulting supernatant was analyzed using an Agilent 6460 Liquid Chromatography-Tandem Mass Spectrometer (LC-MS/MS). Sample preparation was performed in triplicate and analyses done in duplicate. Quantitation was performed using IDMS as described in EPA Method 6800.

For reduced (GSH) and oxidized (GSSG) glutathione analysis, known masses of RBC samples were treated with approximately 8,000 μg g^−1^ N-ethylmaleimide in an inert atmosphere environment while thawing. Each sample was then spiked with known amounts of enriched GSH and GSSG and equilibrated for approximately 30 minutes. The proteins were precipitated out of the samples by adding 1 mL ACN, and then centrifuged at 4°C at 5,000 RCF for 3 minutes. The resulting supernatant was evaporated off under vacuum, and the residue was redissolved in 40 microliters (μL) of water after the addition of 120 μL of ACN for LC-MS/MS analysis. Sample preparation was performed in triplicate with analysis in duplicate. Quantitation was performed using Speciated Isotope Dilution Mass Spectrometry (SIDMS), the other component of EPA Method 6800 [71], that corrected for the interconversion of the glutathione species.

### Behavioral rating scales

The same parent filled out multiple behavioral rating scales within 24 hours of the beginning and end of the cleanroom experience. The rating scales completed included the Social Communication Questionnaire (SCQ) [74], PDD Behavioral Inventory (PDDBI) [75], Aberrant Behavior Checklist (ABC) [76], Gilliam Autism Rating Scale-2 (GARS-2) [77], Autism Treatment Evaluation Checklist (ATEC) [78], and Childhood Autism Rating Scale (CARS) [79]. The SCQ is used to assess the social interactions, language and communication aptitude, and stereotyped and repetitive behaviors of children with an autism spectrum disorder [80]. The PDDBI has multiple domains and composites that measure both adaptive and maladaptive behaviors [81], and the Autism Composite is calculated using selected domains. The total ABC is the summation of five subscales: (I) Irritability, Agitation, Crying; (II) Lethargy, Social Withdrawal; (III) Stereotypic Behavior; (IV) Hyperactivity, Noncompliance; and (V) Inappropriate Speech [76]. The results of these rating scales were recorded and pooled for statistical analysis.

### Statistical analyses

All statistical analyses were performed using the R statistical software package. To investigate an overall cleanroom impact, the n = 10 pre-cleanroom measurements were subtracted from the corresponding post-cleanroom measurements for each variable. Paired t-tests were used to identify whether the observed mean differences deviated significantly from zero. Two separate statistical analyses were performed to investigate the relationship between measurement change and subject age. Preliminary statistical analysis indicated the necessity to divide the entire study population into younger and older cohorts. An independent sample *t*-test was implemented for each variable to assess whether the average difference observed for subjects in the younger cohort, age 5 and under, was significantly different than for subjects in the older cohort, who were age 6 and older. A Pearson correlation between measurement difference and subject age was also computed for each variable.

Replicate analyses were performed for all chemical and elemental tests, allowing for confidence intervals to be used as a means of comparing difference in values. The rating scale and commercial laboratory measurements are single-measurement results and do not provide such statistical power. An independent samples *t*-test was used to determine whether the average of the pre-cleanroom replicate measurements for a particular subject differed significantly from the average of the post-cleanroom replicate measurements for that subject. The fact that funding constraints allowed for only n = 10 subjects is the greatest statistical limitation of this study. This sample size provided relatively weak statistical power (as compared to larger sample sizes) for recognizing small or moderate differences when they exist. This issue, combined with the exploratory nature of this study and the fact that variables within a category are often correlated (as with the rating scale variables), has prompted the authors to consider p-values at or below the 0.05 significance level as indicative of a noteworthy result.

## Results

### Cleanroom classification

The measurements taken during the study showed that the cleanroom met criteria for an ISO Class 5 classification with an average of 1,292 particles 0.5 μm m^−3^. Both the average of the five sampling locations and 95% upper confidence interval (CI) bound for a one sided *t*-test were below the ISO Class 5 limit of 3,520 particles 0.5 μm m^−3^. In comparison to an unmodified, adjacent room, the cleanroom had a 175 fold decrease in the number of 0.5 μm particles.

### Markers of environmental toxin exposure

#### Elemental analysis of blood and hair

**Figure 1:**
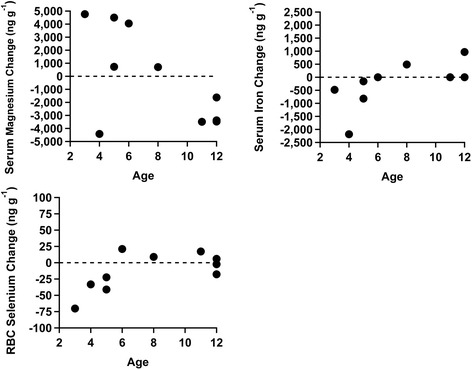
**Changes in serum magnesium, serum iron, and RBC selenium versus age.** Younger children had a greater tendency to raise their serum magnesium than older children as it was negatively correlated with age (p = 0.047). There was a significant positive correlation between the change in iron and age (p = 0.050). There was a near significant positive correlation between age and change in RBC selenium concentration (p = 0.051). There are two points at 0 for age 12 for the serum iron change.

**Figure 2:**
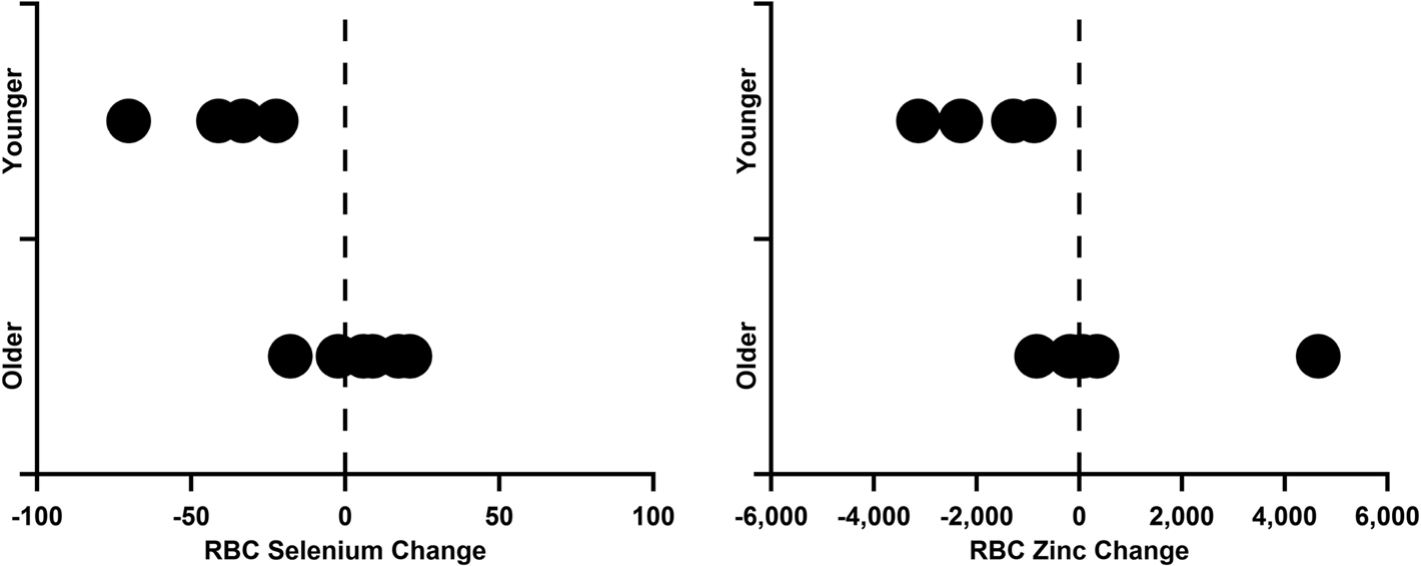
**Elemental analysis results in RBCs for zinc and selenium.** When applying a cutoff at age 5 which separated the younger four children from the older six children, significant decreases were seen for RBC selenium (p = 0.011) and RBC zinc (p = 0.028) in the younger cohort.

Significant results were observed when two types of age analyses were performed (Figures 1 and 2). Significant negative correlation with age was found for changes in serum magnesium (p = 0.047, r = − 0.64). Conversely, significant positive correlation with age was found for changes in serum iron (p = 0.050, r = 0.63). The changes in RBC selenium were nearly significantly positively correlated with age (p = 0.051, r = 0.63). Additional statistical analysis on the age based cohorts indicated that the younger children, ages 5 and under, decreased both their RBC selenium (p = 0.011) and RBC zinc (p = 0.028) to a greater degree on average when compared to the older children, age 6 and older.

No significant changes or age correlations were observed for hair elemental concentrations. It was noted that each of the children had a unique profile of heavy metals in their hair as they displayed varying concentrations of aluminum, antimony, arsenic, cadmium, chromium, lead, manganese, mercury, nickel, and tin in their hair before beginning the study [82].

#### Analysis of organic toxins

**Figure 3:**
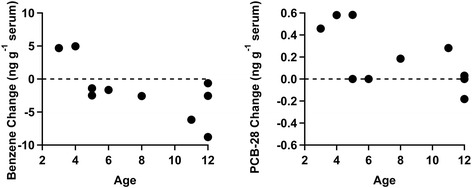
**The results of the differences for benzene and PCB-28 versus age.** The results display a negative correlation with age for each chemical with p = 0.021 for benzene and p = 0.028 for PCB-28.

Detectable quantities of benzene, toluene, and o-xylene were found in all serum samples at, or exceeding, national averages [83]. The change in serum benzene concentration negatively correlated with age (p = 0.021, r = − 0.71) as seen in Figure 3. One child in the older cohort significantly increased toluene by independent samples *t*-test. One child demonstrated a significant drop in o-xylene concentration by independent samples *t*-test. There were no significant differences in VOC concentrations in paired t-tests of pre- and post- findings for the set of ten children.

In every sample in which PCB 28, PCB 52, PCB 101, and PCB 138 exceeded the quantification limit, the serum concentration was greater than the national average for 12–19 year old children [83]. The change in serum PCB 28 negatively correlated with age (p = 0.028, r = − 0.72) as seen in Figure 3. Two children increased PCB 28 concentrations significantly. The concentrations of PCB 52 decreased significantly in two children and increased significantly in two others by independent samples t-tests. No statistically significant changes or correlations were observed when PCB 101 and PCB 138 were detected and quantified, and PCB 101 was detected at the highest average concentration of all of the PCBs measured. In addition, PCBs 153 and 180 were not quantifiable in any of the ten children. There were no significant differences in PCB concentrations in paired t-tests of pre- and post- findings for the entire cohort. Overall, changes in serum benzene and PCB 28 concentrations showed significant negative correlations with age, with the younger children showing the greatest post-cleanroom increase in concentration.

### Markers of oxidative stress

**Figure 4:**
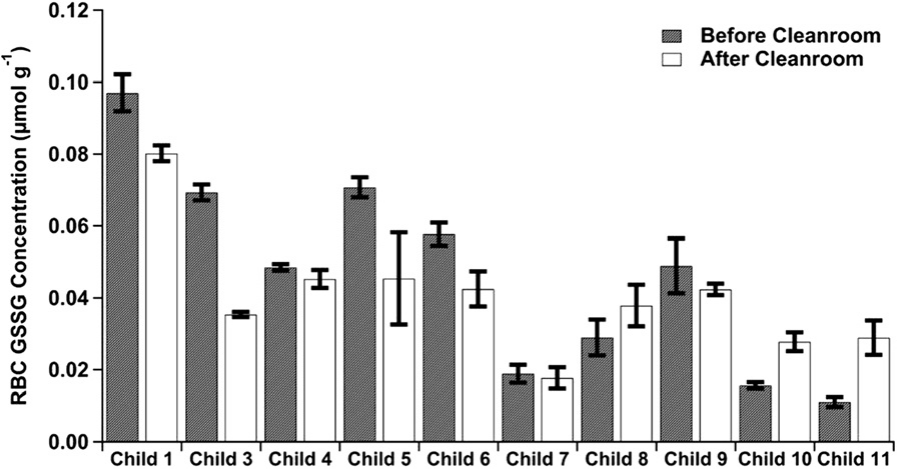
**The before and after results for RBC oxidized glutathione for the ten children (n = 6, 95% CIs).** Seven of the ten children decreased their mean red blood cell oxidized glutathione concentrations, with five of these decreases significant by independent samples *t*-test. Child #2 was unable to participate in the study.

The glutathione results are shown in Table 1. Five children significantly decreased their GSSG after the cleanroom by independent samples *t*-test (Figure 4), four from the older cohort and one from the younger. This younger child also significantly increased GSH, reduced glutathione/oxidized glutathione ratio (GSH/GSSG), and total glutathione/oxidized glutathione (tGSH/GSSG) by independent samples *t*-test. A significant increase of tGSH/GSSG was seen in three children, one younger and two older, along with a significant decrease in tGSH/GSSG ratio in one older child. All ten children displayed the same overall pattern- an increase, even if not statistically significant, of GSH/GSSG was accompanied by a decrease of GSSG or vice versa. No significant differences were observed using paired t-tests comparing pre-and post-measurements of glutathione species of the entire group. In all, five children improved significantly in at least one marker of oxidative stress, with one belonging to the younger cohort.

**Table 1:** **The before and after red blood cell glutathione results (**μ**mol g** ^**−1**^ **) according to EPA Method 6800 (n = 6, 95% CIs)**

	**GSH (**μ**mol g** ^**−1**^ **)**	**GSSG (**μ**mol g** ^**−1**^ **)**	**GSH/GSSG**	**tGSH (**μ**mol g** ^**−1**^ **)**	**tGSH/GSSG**
Child 1 Before	2.06 ± 0.95	**0.0970 ± 0.0051**	21.1 ± 9.0	2.45 ± 0.32	25.3 ± 4.2
Child 1 After	1.87 ± 0.28	**0.0802 ± 0.0022**	23.4 ± 3.9	2.39 ± 0.20	29.8 ± 2.8
Child 3 Before	1.53 ± 0.39	**0.0693 ± 0.0022**	22.3 ± 6.4	2.01 ± 0.21	**29.0 ± 2.9**
Child 3 After	1.00 ± 0.06	**0.0354 ± 0.0007**	28.3 ± 1.9	2.11 ± 0.23	**59.6 ± 6.5**
Child 4 Before	1.58 ± 0.04	**0.0485 ± 0.0009**	32.6 ± 0.4	2.11 ± 0.13	43.4 ± 3.1
Child 4 After	1.81 ± 0.19	**0.0453 ± 0.0025**	40.1 ± 5.4	2.28 ± 0.21	50.3 ± 4.2
Child 5 Before	1.83 ± 0.71	**0.0707 ± 0.0028**	25.9 ± 10.1	2.07 ± 0.29	**29.3 ± 4.2**
Child 5 After	1.77 ± 0.49	**0.0454 ± 0.0128**	39.1 ± 3.4	2.07 ± 0.23	**47.7 ± 11.0**
Child 6 Before	**1.70 ± 0.21**	**0.0577 ± 0.0032**	**29.5 ± 3.5**	2.35 ± 0.29	**40.8 ± 5.4**
Child 6 After	**2.32 ± 0.28**	**0.0425 ± 0.0049**	**54.7 ± 4.8**	2.36 ± 0.26	**55.8 ± 6.0**
Child 7 Before	2.25 ± 1.22	0.0189 ± 0.0025	127 ± 85	2.13 ± 0.20	113 ± 6
Child 7 After	2.32 ± 1.42	0.0177 ± 0.0030	139 ± 96	2.18 ± 0.18	126 ± 24
Child 8 Before	2.35 ± 1.08	0.0290 ± 0.0050	86.3 ± 49.4	2.21 ± 0.38	76.6 ± 10.4
Child 8 After	2.06 ± 0.49	0.0379 ± 0.0058	57.1 ± 24.2	2.25 ± 0.36	61.3 ± 18.0
Child 9 Before	1.72 ± 0.16	0.0489 ± 0.0076	36.0 ± 7.3	1.88 ± 0.16	39.3 ± 8.4
Child 9 After	1.69 ± 0.17	0.0424 ± 0.0016	40.0 ± 4.9	1.86 ± 0.16	44.1 ± 5.3
Child 10 Before	2.08 ± 0.38	**0.0156 ± 0.0009**	134 ± 28	2.75 ± 1.44	179 ± 100
Child 10 After	1.82 ± 0.14	**0.0278 ± 0.0026**	66.1 ± 9.4	2.26 ± 0.55	81.8 ± 20.9
Child 11 Before	2.39 ± 0.62	**0.0111 ± 0.0014**	221 ± 71	2.35 ± 0.42	**217 ± 58.5**
Child 11 After	2.07 ± 0.29	**0.0289 ± 0.0048**	73.9 ± 21.0	2.34 ± 0.31	**83.7 ± 23.2**

The bolded values denote significant difference by independent samples t-tests. Child 2 was unable to participate in the study.

### Markers of immune dysregulation

**Figure 5:**
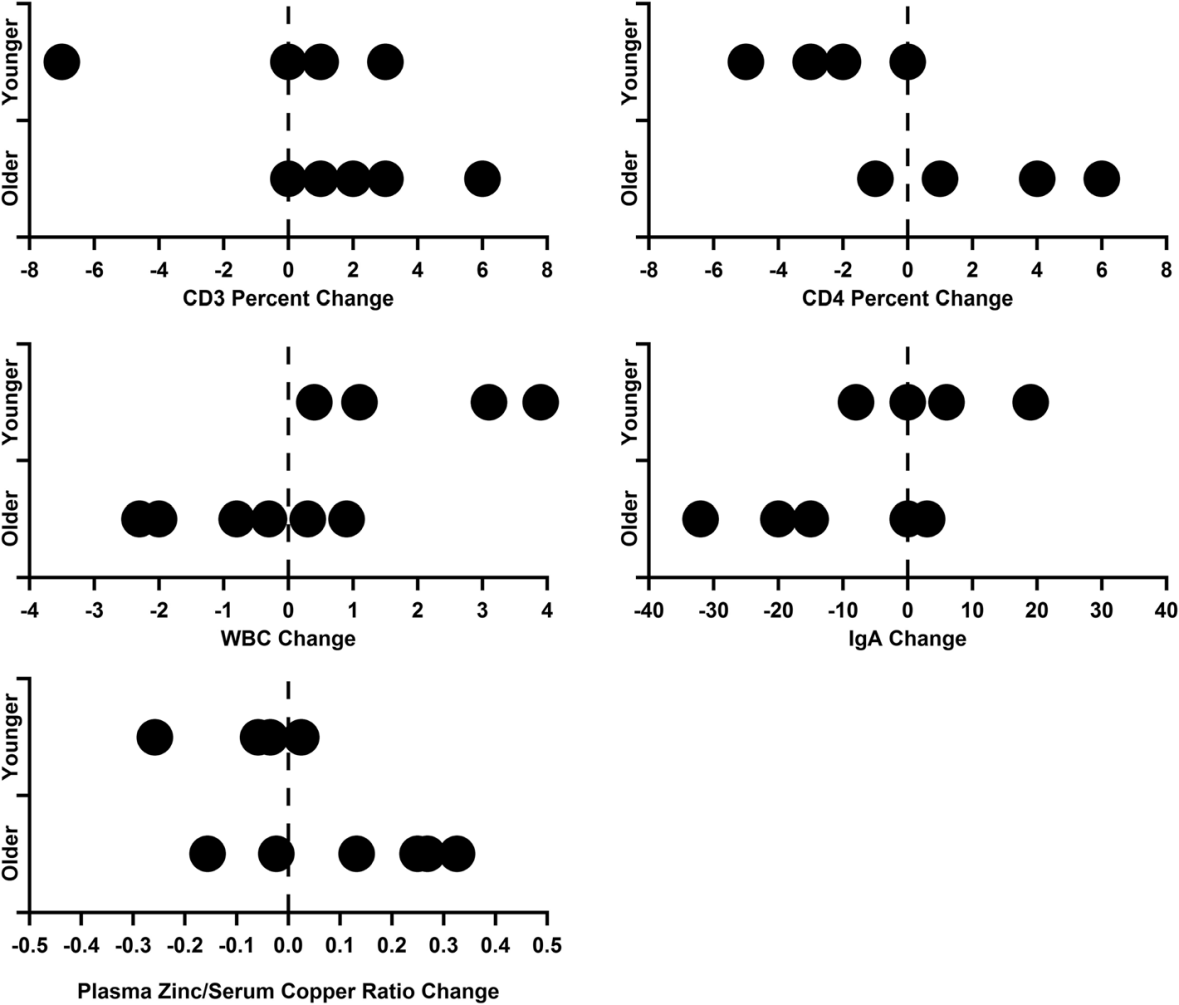
**Results of the clinical laboratory differences for CD3%, CD4%, WBCs, IgA, and zinc/copper ratio.** When applying a cutoff at age 5, separating the younger four children from the older six children, significant decreases were seen for CD3% (p = 0.036) and CD4% (p = 0.028), and a significant increase was seen for WBC (p = 0.031). Trends for increased IgA (p = 0.059) in the younger cohort and elevated zinc/copper ratios (p = 0.061) in the older cohort were present. For CD3% change, two points overlap at +2 in the older cohort. For CD4% change, two points overlap at both – 1 and +1 in the older cohort. In the older cohort for IgA change, two points overlap at – 15.

Significant clinical results for several markers of immune regulation are summarized in Table 2. When separated into cohorts, the younger children significantly decreased their CD3% (p = 0.036) and CD4% (p = 0.028) compared to the older children. Total white blood cell (WBC) count significantly increased in the younger children and decreased in the older children (p = 0.031). The younger cohort tended to increase their IgA levels, while the older cohort tended to lower their IgA levels (p = 0.059). The clinical laboratory plasma zinc/serum copper ratio exhibited a tendency to increase in the older six children compared to the four younger children (p = 0.061). The results for these markers of immune dysregulation for the two cohorts are shown in Figure 5.

**Table 2:** **Statistically significant changes and trends in the commercial laboratory results of mean differences between age groups when a cutoff is applied separating children ages 3–5 from children ages 6–12**

**Commercial laboratory variable**	**Mean change younger cohort**	**Mean change older cohort**	**p-value**
CD3%	−2.75	2.33	0.036
CD4%	−2.50	1.67	0.028
WBC	2.13	−0.70	0.031
IgA	4.25	−13.17	0.059
Zinc/Copper Ratio	−0.08	0.13	0.061

### Behavioral rating scales

**Figure 6:**
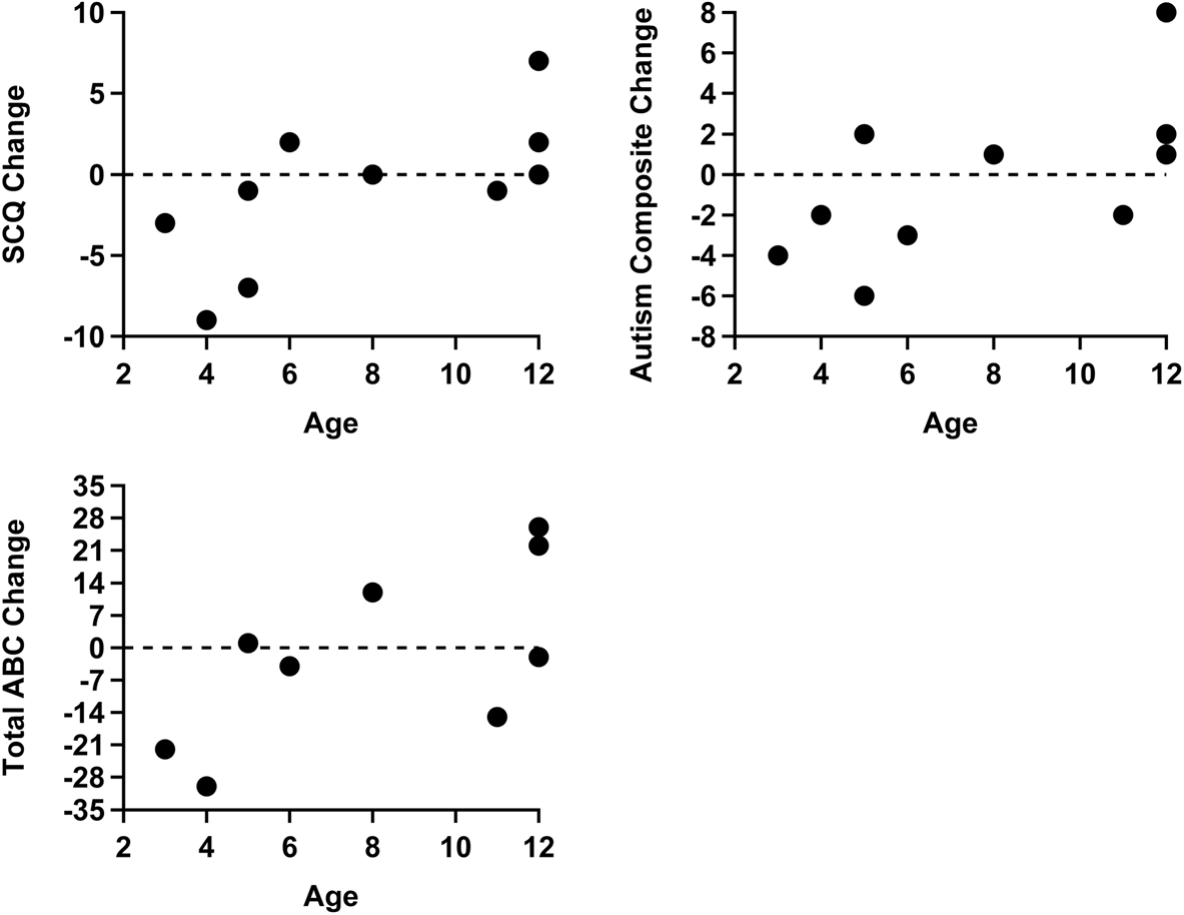
**Differences for the SCQ, Autism Composite of PDDBI, and total ABC rating scales versus age.** The results display significance or near significance for each rating scale with a p-value of 0.030 for SCQ, 0.046 for Autism Composite, and 0.054 for total ABC. Two points overlap at age 5 with a difference of +1 for total ABC.

The before and after cleanroom rating scale results for all 10 children are summarized in Table 3 with significant correlation coefficients (r) and p-values listed in Table 4. The younger cohort demonstrated significant decreases in SCQ means (p = 0.025). The changes in SCQ versus age were also significant, with a positive correlation (r = 0.68, p = 0.030) seen in Figure 6. Additional positive correlations with age were found in the changes in Autism Composite of the PDDBI (r = 0.64, p = 0.046) and the Sensory/Perceptual Approach Behaviors (SENSORY) subscale of the PDDBI (r = 0.64, p = 0.045). The Receptive/Expressive Social Communication Abilities Composite (REXSCA/C) showed significant negative correlations with age (r = −0.66, p = 0.037), which indicates language improvement in the younger cohort. None of the other domains or composites of the PDDBI produced significant results. The change in the total ABC by age of the ten children was nearly statistically significant with a p-value of 0.054 (r = 0.62). Furthermore, three of the five subscales of the ABC were significantly positively correlated with age (p = 0.034 for subscale III, p = 0.024 for subscale IV, and p = 0.035 for subscale V). The two remaining subscales of ABC showed no significant correlations.

**Table 3:** **Child number, age, and rating scale results for the children who experienced the cleanroom sleeping environment**

**Child number**	**Age**	**ADOS composite**	**SCQ**		**Autism composite (PDDBI)**		**Total ABC**		**Total ATEC**		**CARS**		**Autism index (GARS-2)**	
		**Before**	**Before**	**After**	**Before**	**After**	**Before**	**After**	**Before**	**After**	**Before**	**After**	**Before**	**After**
**1**	6	17	22	24	57	54	38	34	51	60	36	34	85	91
**3**	12	13	20	27	76	84	92	118	85	96	47	49.5	119	126
**4**	12	15	28	30	73	75	69	91	101	96	46.5	48	106	117
**5**	11	14	25	24	61	59	65	50	59	44	40	43	100	103
**6**	5	14	27	20	50	44	12	13	62	58	34	36.5	79	74
**7**	8	16	30	30	65	66	116	128	95	108	51	50.5	112	115
**8**	5	12	21	20	45	47	13	14	55	54	33.5	33	64	81
**9**	4	16	23	14	41	39	57	27	55	57	39	26.5	82	64
**10**	3	17	26	23	57	53	64	42	52	59	39	38	94	87
**11**	12	14	13	13	41	42	5	3	51	49	21	22	76	85

Child 2 was unable to participate in the study. Children 3 and 4 and children 6 and 8 were identical twins.

**Table 4:** **Statistically significant difference correlations comparing child age and difference in rating scale scores**

**Rating scale**	**r**	**p-value**
SCQ	0.68	0.030
PDDBI Autism composite	0.64	0.046
Total ABC	0.62	0.054
ABC III	0.67	0.034
ABC IV	0.70	0.024
ABC V	0.67	0.035
PDDBI REXSCA/C	−0.66	0.037
PDDBI SENSORY	0.64	0.045

The correlations and p-values are shown for each significant variable.

There were no significant differences in rating scales results in the paired *t*-test analyses of pre- and post- results. The GARS-2, ATEC, and CARS produced no statistically significant results for change or age correlation. In total, six scales, subscales, domains, or composites used as markers of ASD behavioral presentation agreed in finding a statistically significant correlation with age between pre-and post-cleanroom ratings. Of note, each of the four younger children improved on the SCQ scale. Of these four, two raised their GSH/GSSG ratio, with one significant by independent samples *t*-test.

### Twins

The study contained two sets of identical twins, one younger and one older set. Children 6 and 8 were five-year-old females. Child 6 significantly improved on four out of five markers of oxidative stress seen in Table 1 (GSH, GSSG, GSH/GSSG, and tGSH/GSSG). This improvement in oxidative stress was mirrored by a better performance in the rating scales SCQ and PDDBI; the subscales ATEC I, ATEC II, ABC I, ABC II, ABC V; GARS-2 Communication, Social Interaction, and Autism Index; and all of the maladaptive domains and composites and the Autism Composite of the PDDBI (Table 3). The corresponding twin, Child 8, did not improve on any markers of oxidative stress and, likewise, did not improve performance on rating scales, subscales, composites, and indexes.

Children 3 and 4 were twelve-year-old male identical twins. Both children raised their GSH/GSSG, with Child 4 showing greater improvement. This disparity in improvement was mirrored in better performance for Child 4 on many rating scales, subscales, and composites. Child 4 demonstrated better performance on the PDDBI, CARS, and SCQ; ATEC total and subscales; ABC total, I, and IV; Communication subscale of GARS-2; and the SENSORY, Social Pragmatic Problems, Semantic/Pragmatic Problems, Arousal Regulation Problems, Specific Fears, Aggressiveness, Social Approach Behavior domains along with the Repetitive, Ritualistic, and Pragmatic Problem Behaviors, Approach/Withdrawal, and Autism Composites of the PDDBI [82]. In both sets of identical twins, the child with the larger improvement in markers of oxidative stress showed the greater improvement in behavioral rating scales following the study.

## Discussion

During this study ten children with ADOS certified autism slept in a hospital cleanroom for a two week period. The cleanroom maintained ISO Class 5 certification, which minimized the nighttime exposure of both children and parents to heavy metals and VOCs. Given the emergent data linking exposure to environmental toxins with autism etiology, experiencing a cleanroom environment during sleep was hypothesized to begin the process of detoxification by mobilizing heavy metals and chemicals into the blood and hair.

Five children significantly lowered their GSSG concentrations in RBCs, the component of blood with the highest glutathione concentration [84]. The decrease in GSSG concentrations could indicate a reduction of oxidative stress, suggesting an increased ability to eliminate inflammation, prevent infections, and detoxify environmental toxins [39]. Three children raised their tGSH/GSSG ratio, an improvement in an indicator of the balance of glutathione species [38], which was driven by a decrease in GSSG. In all ten children, an inverse relationship was observed between the change in glutathione ratios, tGSH/GSSG and GSH/GSSG, and the change in GSSG concentration. High GSH/GSSG ratios preserve the necessary reducing environment inside of cells [85]; abnormally low GSH/GSSG ratios may indicate an inadequacy of glutathione reductase [39]. Numerous studies have noted the multiple functions regulated by the redox balance of GSH/GSSG [33,39,86]. Decreases in GSSG may have been the result of improved transulfuration reducing the demand for adenosine [38], the nucleoside that prevents oxidative damage [87,88].

The younger cohort showed significantly greater decreases in RBC zinc and selenium concentrations than the older cohort. The younger children may have moved selenium and zinc more rapidly from red blood cells to tissues, increasing metalloprotein functioning more than the older children. In addition, serum magnesium changes were significantly negatively correlated with age, increasing in concentration in the younger children. Magnesium is a critical micronutrient that is essential in the maintenance of the calcium/magnesium flow across neuronal cell membranes [89]. It also plays a key role in enzymes related to ATP and nucleic acid metabolism [90]. Proper brain development and homeostasis require magnesium resulting from copper-zinc superoxide dismutase activity [91]. When given as a supplement, magnesium has been shown to have a calming effect on the behavior of children and adults [92,93], which may explain the link between increased serum concentrations and improved behavioral presentations observed in this study. Additionally, iron concentration changes correlated positively with age. The younger children decreased and the older children increased their respective concentrations. Iron is critical for erythrocyte production and the promotion of oxygenation of tissues; its effects on metabolism depend on local concentration [94]. At higher levels, though, iron can cause an excessive production of reactive oxygen species, digestive problems, vomiting, and the alteration of other closely related elements such as zinc, copper, and calcium [95]. The observed positive age correlation in serum iron may be explained by a faster upregulation in the metallothionein system of the younger children. This upregulation would lead to an increased need for erythrocyte production to enhance oxygen distribution to tissues, depleting serum iron. Hair selenium concentrations tended to correlate negatively with age. The younger children had either increases or small decreases in their hair selenium concentrations compared to the older ones. The increased release of selenium in the hair in the younger children may have been secondary to increased absorption of selenium. Significant changes in hair concentrations of elements may have required a longer period of exposure to cleanroom conditions.

Prior to exposure to the cleanroom, VOCs and PCBs were quantified at concentrations above the national averages for adolescents [83] in many of the children. Out of the ten participants, eight decreased mean serum benzene, six increased mean serum toluene and five increased serum xylene, although none significantly. One possible explanation for these results is that, throughout the study, VOCs were mobilized from tissues into the serum at varying rates. In this scenario, benzene would be mobilized and released from the body first, leading to decreased serum concentrations. Toluene and o-xylene, however, could require more time to mobilize and result in the tendency for increased serum concentrations following the two week exposure to the cleanroom. Likewise, mean PCB-28 concentrations rose in six out of seven children, which significantly negatively correlated with age, with three children falling below the analytical quantification limit. This result is consistent with the hypothesis of the cleanroom beginning the process of mobilization of PCB-28 from tissue to serum, but requiring more time for elimination from the serum.

Following exposure to the cleanroom environment, the children demonstrated changes in markers of immune system functioning. These changes indicate a decrease of T cell subtypes instructing B cells to make antibodies. Notably, the younger children decreased CD3% (total T cell percent) and CD4% (total helper T cell percent) significantly more than the older cohort. This data provides evidence that the cleanroom may have decreased dysregulation of the immune system, especially in the younger children. B cells promote immunoglobulin synthesis and are regulated by CD4 cells. The observed decrease in CD4% may have lessened cross reactive antibody production in the younger children, leading to a brief period of decreased brain inflammation. This is one possible explanation of the significant, but transient, improvement seen in the behavioral rating scale performances of the younger cohort.

Significant changes in before and after cleanroom results were seen for rating scale performance when compared with age. Specifically, the younger children in the study tended to improve on the behavioral rating scales SCQ, sub-domains of the PDDBI, and ABC while the older children tended to do slightly worse on these rating scales. The PDDBI was created specifically to evaluate how children with pervasive developmental disorders respond to a treatment, with the unique capability to standardize results by age [81]. In contrast with other scales and indices, the PDDBI assesses both adaptive and maladaptive behaviors, giving a quantitative definition to positive improvements. A significant age-correlation was noted in the Autism Composite of the PDDBI, with the younger children showing greater improvements in non-verbal communication, repetitive behaviors, social interaction, and spoken language. The SENSORY domain of the PDDBI produced a significant negative correlation with age indicating more improvement of nonverbal communication in the younger children compared to the older children. The negative correlation with age on the REXSCA/C demonstrates significantly more improvement in receptive and expressive language attainment in the younger children compared to the older children. The improvements on the total ABC, designed to assess treatments for individuals with intellectual disabilities [76], were primarily driven by the significant improvements on the stereotypical behavior, hyperactivity, and speech subscales. One possible explanation of these rating scale results is that the younger children may have had less of an initial body burden of heavy metals and chemical toxicants, allowing the brief two week period of sleeping in the cleanroom to have a slightly more positive behavioral effect. The older children, who may have had a larger body burden of toxicants initially, may have just begun the process of mobilization of chemicals and metals as the study ended, resulting in worse behavioral result changes in relation to the younger children. Younger children may have felt an extra closeness to their parents during the study, leading to positive behavioral change. The interpretation of this study is limited by the small sample size, lack of Bonferroni correction for multiple comparisons, and absence of a control group. However, age-correlated findings involving changes in elemental, glutathione, and immune markers observed in this study provide support for physiological change driving the behavioral improvements in the younger children.

One of the unique results of this study involved the responses of two sets of identical twins and the manner in which changes in glutathione species corresponded with behavioral rating scale results. In both sets, one twin demonstrated greater improvement in oxidative stress, as evidenced by glutathione markers, and corresponding greater improvement in behavioral rating scale scores. The GSH/GSSG ratio is a critical biomarker for determining healthy functioning of the methylation system in children with autism [96]. In the 5-year-old female identical twin pair, the twin who raised her GSH/GSSG ratio also performed better on two behavioral rating scales, the SCQ and PDDBI. Notably, this female twin displayed the greatest improvement of oxidative stress in the study, significantly lowering GSSG and increasing GSH, tGSH/GSSG, and GSH/GSSG values. In the older male identical twin pair, both children worsened on the behavioral scales, but the twin with greater GSH/GSSG ratio improvement displayed less worsening on the SCQ and PDDBI. Despite identical home settings and genomes, it was found in both sets that each twin responded uniquely to sleeping in the cleanroom, displaying varying degrees of change in GSH/GSSG levels and behavioral rating scale performance. These differences suggest that still unknown epigenetic mechanisms mediated the varying results between the twins in the study. This study provides further support to the assertion of a relationship between glutathione biomarkers and behavioral rating scores in children with autism.

## Conclusions

In this study, ten children with ADOS certified autism spent two weeks sleeping in a cleanroom. The overall pattern of results indicates greater improvements in immune dysregulation and behavior in the younger children, age 5 and under. Half of the children significantly decreased their oxidative glutathione concentrations in addition to improving other glutathione markers of oxidative stress. The relationship between GSH/GSSG and the identical twins’ behavioral performances, the opposite movements of serum iron and magnesium compared to age, and the tendency for the younger children to reduce their CD3% and CD4% all suggest the possibility of a significant physiological role for the cleanroom in explaining some of the behavioral changes. Younger children performed better on rating scales after their two week exposure, while the older children performed slightly worse on rating scales. Psychological issues, such as greater attachment towards the parent in the cleanroom, may explain some of this difference; however, the noted biological differences in how the younger children performed versus older children suggest that the cleanroom may have had a more than psychological effect. The children only slept or stayed for approximately ten hours a night in a cleanroom for two weeks; yet, significant changes in markers of oxidative stress, immune dysregulation, and behavior were observed. In addition, an age correlated difference in detoxification physiology is suggested by the results. At the age of 6, a significant difference tended to occur in the direction of change for some elemental nutrients (iron, magnesium, selenium, and zinc), organic toxicants (benzene and PCB 28), and immune markers (CD3% and CD4%). The younger children may have started their detoxification process faster or had less to detoxify, thus explaining their significant improvement in global rating scales (SCQ, PDDBI, and ABC) compared with the older children. The differential response to the cleanroom by the identical twins suggests that epigenetic changes may be playing a role in affecting responses to a detoxifying environment. Interpretation of this prospective cohort study is limited by the lack of a control group and small sample size. The exploratory nature of the study supported not performing Bonferroni correction and the use of a p value of 0.05 for significance. The performance of a controlled study of 24 hour per day cleanroom exposure for children with autism appears to be an appropriate next step, given the physiologic changes from the limited exposure to a cleanroom environment noted in this study.

## Abbreviations

ABC: Aberrant Behavior Checklist
ACN: Acetonitrile
ADOS: Autism Diagnostic Observation Scale
AIT: Applied Isotope Technologies
ASD: Autism spectrum disorder
ATEC: Autism Treatment Evaluation Checklist
CARS: Childhood Autism Rating Scale
CD3%: Total T cell percent
CD4%: Helper T cell percent
CD4/CD8: Helper/suppressor ratio
CI: Confidence interval
GARS-2: Gilliam Autism Rating Scale-2
GSH: Reduced glutathione
GSH/GSSG: Reduced glutathione/oxidized glutathione ratio
GSSG: Oxidized glutathione
HEPA: High efficiency particulate air
IDMS: Isotope Dilution Mass Spectrometry
IRB: Institutional Review Board
ISO: International Organization for Standardization
LC-MS/MS: Liquid chromatography-tandem mass spectrometer
mg: Milligram
NK: Natural killer
PCB: Polychlorinated biphenyl
PDDBI: PDD Behavioral Inventory
r: correlation coefficient
RBC: Red blood cell
RCF: Relative centrifugal force
REXSCA/C: Receptive/Expressive Social Communication Abilities Composite
SCQ: Social Communication Questionnaire
SENSORY: Sensory/Perceptual Approach Behaviors
SIDMS: Speciated Isotope Dilution Mass Spectrometry
TCI: The Children’s Institute
tGSH: Total glutathione
tGSH/GSSG: Total glutathione/oxidized glutathione ratio
μg g^−1^: Microgram gram^−1^
μL: Microliter
μm: Micrometer
VOC: Volatile organic compound
WBC: White blood cell
